# Comparative Efficacy and Acceptability of Antidepressants in Parkinson's Disease: A Network Meta-Analysis

**DOI:** 10.1371/journal.pone.0076651

**Published:** 2013-10-02

**Authors:** Jinling Liu, Jiangchuan Dong, Lei Wang, Ying Su, Peng Yan, Shenggang Sun

**Affiliations:** 1 Department of Neurology, Union Hospital, Tongji Medical College, Huazhong University of Science and Technology, Wuhan, Hubei, China; 2 Department of Traditional Chinese Medicine, Chongqing Medical University, Chongqing, China; 3 Department of Neurology, Weifang People's Hospital, Weifang, Shandong, China; Karolinska Institute, Sweden

## Abstract

**Background:**

Depression is a common non-motor symptom in patients with Parkinson's disease (PD). There are many kinds of antidepressants being used, such as tricyclic antidepressants (TCAs), selective serotonin reuptake inhibitors (SSRIs), serotonin and norepinephrine reuptake inhibitors (SNRIs), and Dopamine agonists which are suggested as alternative antidepressants for the treatment of depression in PD. Which one should we choose first? Literatures have shown inconsistent results.

**Methods:**

We conducted a network meta-analysis of randomized controlled trials to compare the efficacy and acceptability of therapeutic methods for the treatment of depression in Parkinson's disease.

**Results:**

We used the odds ratios (OR) as effect size firstly and the results indicated no statistical significance between each compared intervention. Then we used the logarithm of the individual odds ratios as effect size. With efficacy of TCAs as the standard of comparison, the degree of incoherence (a measure of how closely the entire network fits together) was small (ω =  4.824827e-05). The logor were: SSRIs −0.69 (95% CI −1.28– −0.10); Pramipexole −0.73 (−1.71– −0.26); Pergolide −1.97 (−3.67– 0.27); SNRIs −0.86 (−1.86– 0.15); Placebo −1.24 (−1.99– −0.50). With Placebo as the standard of comparison, the logor were: TCAs 1.24 (0.50– 1.99); SSRIs 0.55 (−0.03– 1.13); Pramipexole 0.51 (−0.12– 1.15); Pergolide −0.73 (−2.25– 0.80); SNRIs 0.38 (−0.42– 1.19); TCAs, pramipexole, pergolide and SNRIs showed better profile of acceptability, leading to significant fewer discontinuations than that of SSRIs.

**Conclusions:**

There is insufficient evidence to support antidepressant efficacy for SSRIs, pramipexole, pergolide and SNRIs. TCAs might be the best choice when starting antidepressant treatment in patients of Parkinson's disease because it has the most favorable balance between benefits and acceptability, followed by pramipexole and SNRIs, SSRIs might be the last choice.

## Introduction

Depressive disorders as well as depressive symptoms are common in Parkinson's disease (PD) and are important factors affecting quality of life. There are many pharmaceutical therapy options for depression in Parkinson's disease (PD): such as TCAs, SSRIs, SNRIs and Dopamine agonists which are being suggested as alternative antidepressants for these patients. A Cochrane Review published in 2003 concluded that there was insufficient data on the effectiveness and safety of any antidepressant therapies in PD to allow recommendations for their use [1]. Results of a meta-analysis in 2005[2] suggested a very large effect for both active treatment and placebo in PD depression, but no difference between the two. Nevertheless, antidepressants are apparently widely used. SSRIs are now the most commonly prescribed antidepressants in patients with depression in PD[3], while a meta-analysis in 2010 by Petros Skapinakis and colleagues suggested that there was insufficient evidence to reject the null hypothesis of no differences in efficacy between SSRIs and placebo in the treatment of depression in PD. The comparison between SSRIs and TCAs didn't show statistical difference [4]. However, another study by Menza [5] suggested that the SSRI paroxetine CR was not superior to placebo in patients with PD and depression and might be inferior to nortriptyline. A recent review [6] by Klaus Seppi et al. considered that pramipexole was efficacious for the treatment of depressive symptoms in PD, whereas there was insufficient evidence regarding to pergolide. Which one is our best choice? Literatures have shown inconsistent results.

Traditional meta-analyses can just do direct comparison. Statistical techniques have been developed to establish the relative efficacies of different treatment strategies even when these treatments have not been directly compared [7]. The so-called network meta-analysis complements traditional meta-analyses and systematic reviews. Faced with multiple treatment options, these analyses provide the clinician, the guideline developer, or health care authorities with some hierarchy of effect when different competing interventions are considered or when direct evidence is lacking [8]. Veazey [9] said there were few randomized controlled trials (RCTs) of these treatment options. As time goes by, we have more RCTs. With the new approach, we hope we can give an answer to the above question.

The aim of this article is to systematically review the efficacy and acceptability of antidepressants used in PD patients by network meta-analysis.

## Methods

We undertook a systematic review to identify randomized clinical trials of antidepressant treatments that were published in English before February 2013. We searched PubMed, MEDLINE, Embase, and the Cochrane Collaboration's Database, using the following MeSH terms: “Parkinson's disease, Parkinson disease, depression, antidepressants, randomized controlled trials and meta-analysis”. Additionally, we reviewed the reference lists of all the meta-analyses [4], [10]–[12] and publications for other potential data sources. Study participants were required to have a clinical diagnosis of idiopathic Parkinson's disease and also a clinical diagnosis of depression (as defined by the authors of the trials). Specific depression assessments as primary or secondary outcomes were necessary. The reference lists of all trial reports were examined to identify any additional publications not found in the original search. Data extraction was performed independently by two of the authors and checked by another. To assess the methodological quality of included trials we used the criteria for quality assessment recommended by the Cochrane Collaboration Handbook [13] which are mainly focused on descriptions of sequence generation, allocation concealment, blinding, completeness of outcome data, selective outcome reporting and other potential sources of bias.

### Outcome measures

Response and dropout rates were chosen as primary outcomes, being the most consistently reported estimates of treatment efficacy and acceptability. We defined response as the proportion of patients who had a reduction of at least 50% from the baseline score on the scales for depression assessment, such as Hamilton depression rating scale (HDRS), Montgomery–Asberg depression rating scale (MADRS), BDI total score and others or who scored much or very much improved in the Clinical Global Impression Scale (CGI). When a trial had reported results from several scales, we used the HDRS as the first choice, followed by MADRS and CGI. We used the dichotomous response as our primary outcome but not the reduction in the severity of symptoms measured as a continuous outcome, because we think that results are more readily interpretable from a clinical perspective. We defined treatment discontinuation(acceptability) as the number of patients who terminated the study early for any reason during the treatment (dropouts).

### Statistical analysis

We first performed a traditional meta-analysis to yield the Mantel-Haenszel odds ratio. If one trial with zero events in both groups, the event rate had been artificially inflated by adding 0.5; if each one trial with zero in both groups, the data would be excluded. Heterogeneity between trials was quantified with the I^2^ and H measure. If heterogeneity was moderate or great, we did meta-analysis by comparing the same interventions with a random-effects model [14]. We did the analyses using Stata version 14.

We did a network meta-analysis using random-effects model in R2WinBUGS [15]. We modeled the binary outcomes in every treatment group of every study, and specified the relations among the odds ratios (ORs) across studies to make different comparisons [16]. This method combines direct and indirect comparison for any given pair of treatments. Consistency test was also performed. Analyses were performed in the statistical package R 2.15.3.

We also looked at the comparative efficacy and acceptability among the antidepressant drugs. We expressed the results using TCAs as reference drug, as they were the longest applicated antidepressants for clinicians. We did this network meta-analysis using the one-line program published by Lumley [17]. Based on the extracted data, we didn't have enough data about adverse effect to do another meta-analysis, so side effects were presented in a descriptive way.

## Results

### Included trials

**Figure 1:**
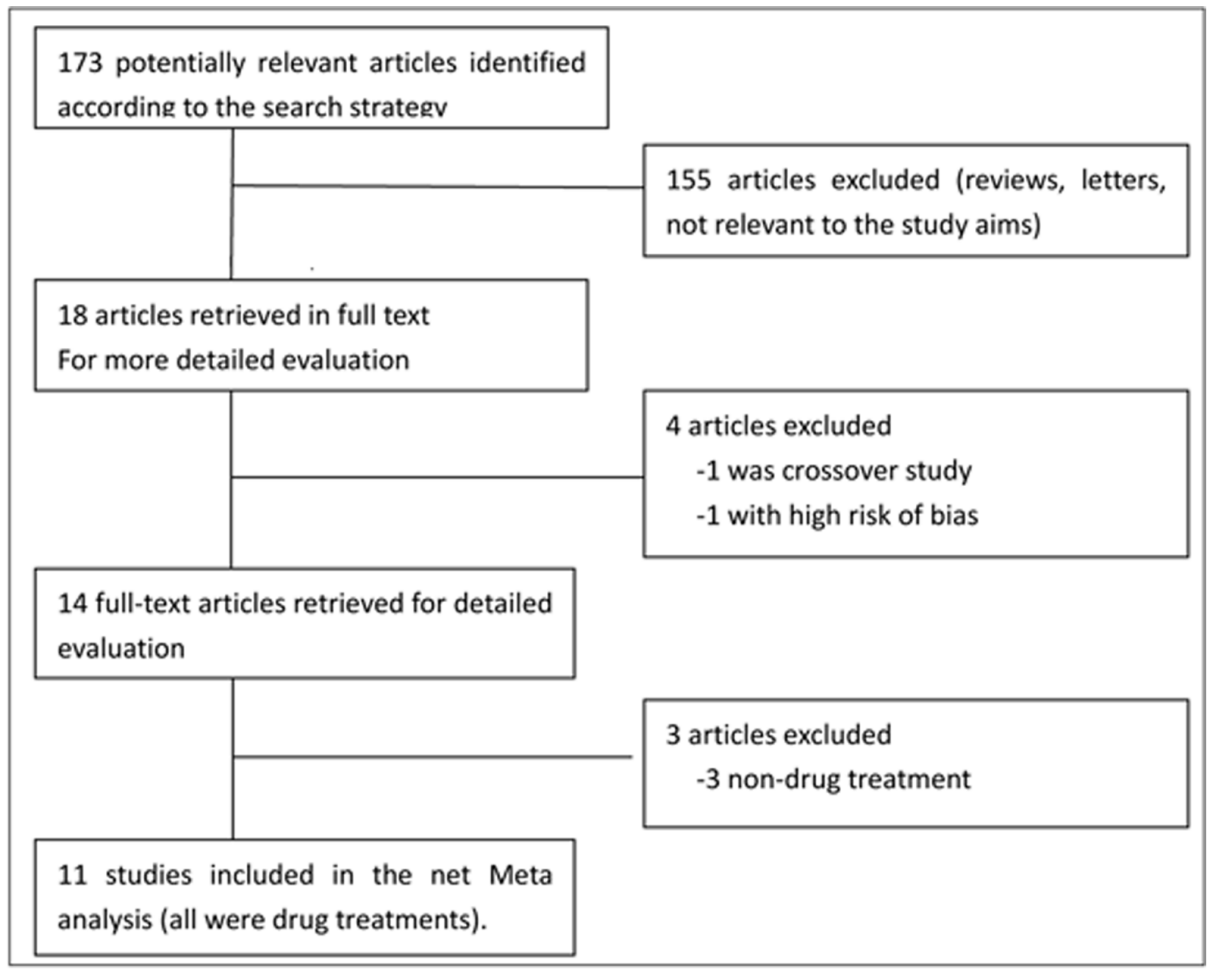
Flow diagram of the study.

**Figure 2:**
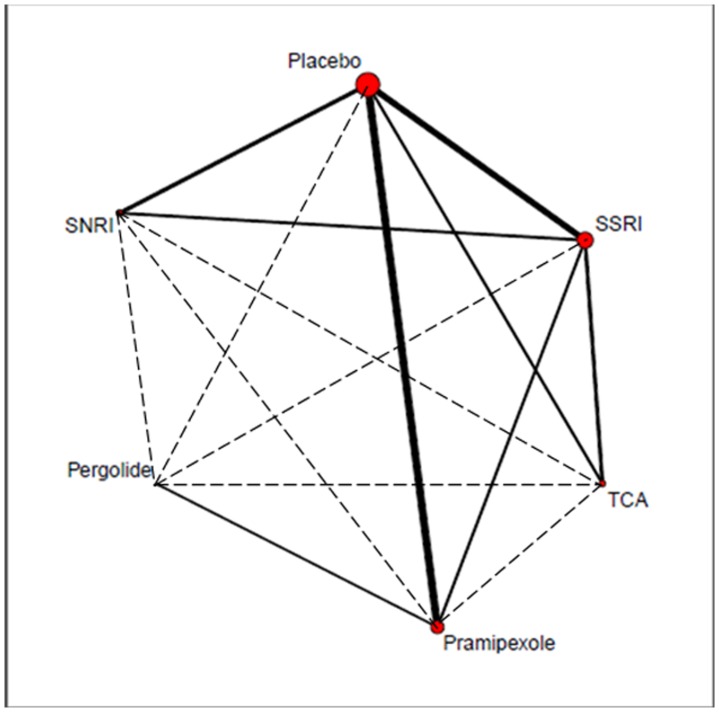
Network of clinical trials of antidepressant drugs in Parkinson's disease. Solid lines represent direct comparison trials, and dashed lines represent indirect comparisons. The size of red circle meant the total patients number of each treatment, the larger circle, the bigger number. The line's thickness represented the total patients' number of pair-wise comparisons.

The electronic searches yielded 173 potentially relevant studies. We excluded 155 reports that did not meet eligibility criteria (figure 1). Overall, we used 11 trials from 1986 to 2013 for the multiple-treatments meta-analysis. Detailed characteristics of all studies included in the meta-analysis are listed in Table S1 [5], [18]–[27]. Three trials were three-arm trials. The study by Antonini et al. [23] compared standard dose of sertraline to a very low dose of amitriptyline (25 mg/day). This dose is not normally considered to have antidepressant potency and a meta-analysis of the efficacy of low versus standard dose amitriptyline generally identified papers with doses not less than 37.5 mg/day [28]. Therefore one can consider this dose as an active placebo with the added advantage of a diminished unblinding effect [4], [29]. The study by Avila et al. [30] was excluded as treatment responders were not available. The trial by Andersen et al. [12] compared nortriptyline with Placebo performed in two neurological outpatient departments in Denmark. It was excluded for design was a double-blind crossover study [27]. Figure 2 showed the network of eligible comparisons for the multiple-treatments meta-analysis.

In most studies adverse effects were reported spontaneously and verbally to the investigators or via questionnaires. All drug treatments were generally well tolerated. Common side effects for the SSRIs group included nausea, fatigue/asthenia and diarrhea, for the TCAs group dry mouth, somnolence, constipation and orthostatic hypotension and for SNRIs group insomnia, somnolence, constipation, sexual dysfunction, headache and hypertension. Nortriptyline could increase the P–R interval, QRS duration, and Q–Tc interval and had been associated with cardiac arrhythmias. Insomnia was reported significantly less frequently in the paroxetine group than in the venlafaxine extended release and placebo groups.

The most common treatment-emergent adverse event of DA agonist was nausea, followed by headache, dizziness, and somnolence, aggravation of dyskinesias, orthostatic hypotension, and hallucinations. There was a significant difference between pramipexole and pergolide groups only with regard to the total number of patients who experienced sleep disturbances at the 5% significance level.

There were three serious adverse events in Richard research. One subject in the placebo group was hospitalized after four days of intermittent chest pressure; however, this subject had completed the study. Another subject in the placebo group was hospitalized for a bowel obstruction, and this subject also had completed the study. One subject in the paroxetine group had frequent, significant ventricular ectopy with >13,000 premature ventricular contractions detected during 24-hour Holter monitoring, and the subject had withdrawn from the trial and treatment assignment was disclosed. Barone in 2010 showed six patients in the pramipexole group and six in the placebo group had serious adverse events (no detailed description). Research by Weintraub had four serious adverse events requiring hospitalization occurred during the study, two in atomoxetine-treated patients (suicide ideation several weeks after study termination in one patient, and exacerbation of congestive heart failure in another) and two in placebo-treated patients (chest pain and possible anxiety attacks in one patient, and urosepsis in another). None of the serious adverse events were thought to be related to the study treatment. Menza reported three serious adverse events: one patient on paroxetine CR was hospitalized for fainting—no cause was discovered— and one patient on placebo had a severe worsening of rigidity due to a Parkinson medication change and another patient on placebo had delirium. Other researches didn't found serious adverse events.

### Direct comparisons

**Figure 3:**
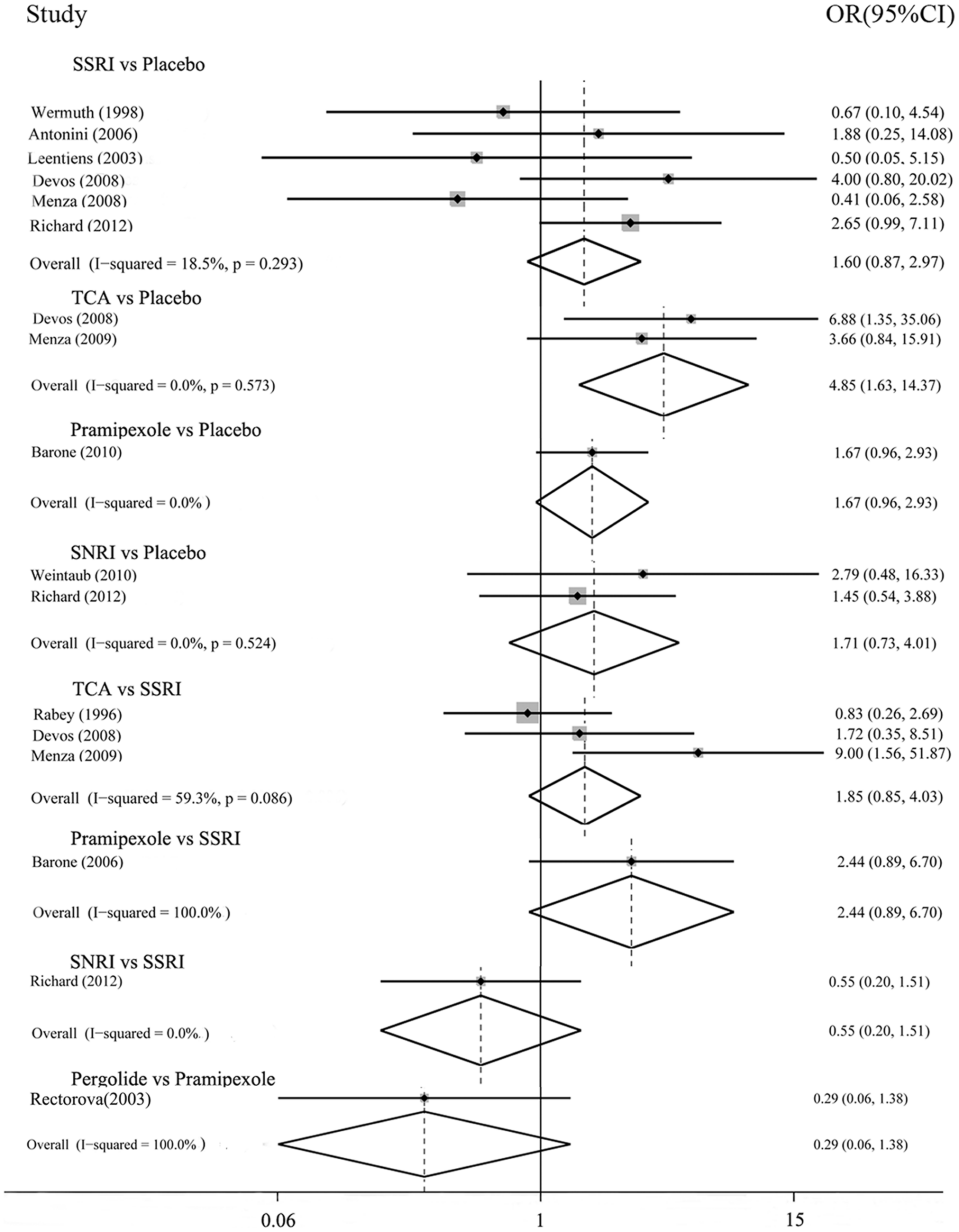
Efficacy: direct comparisons between each pair of antidepressant treatment.

**Figure 4:**
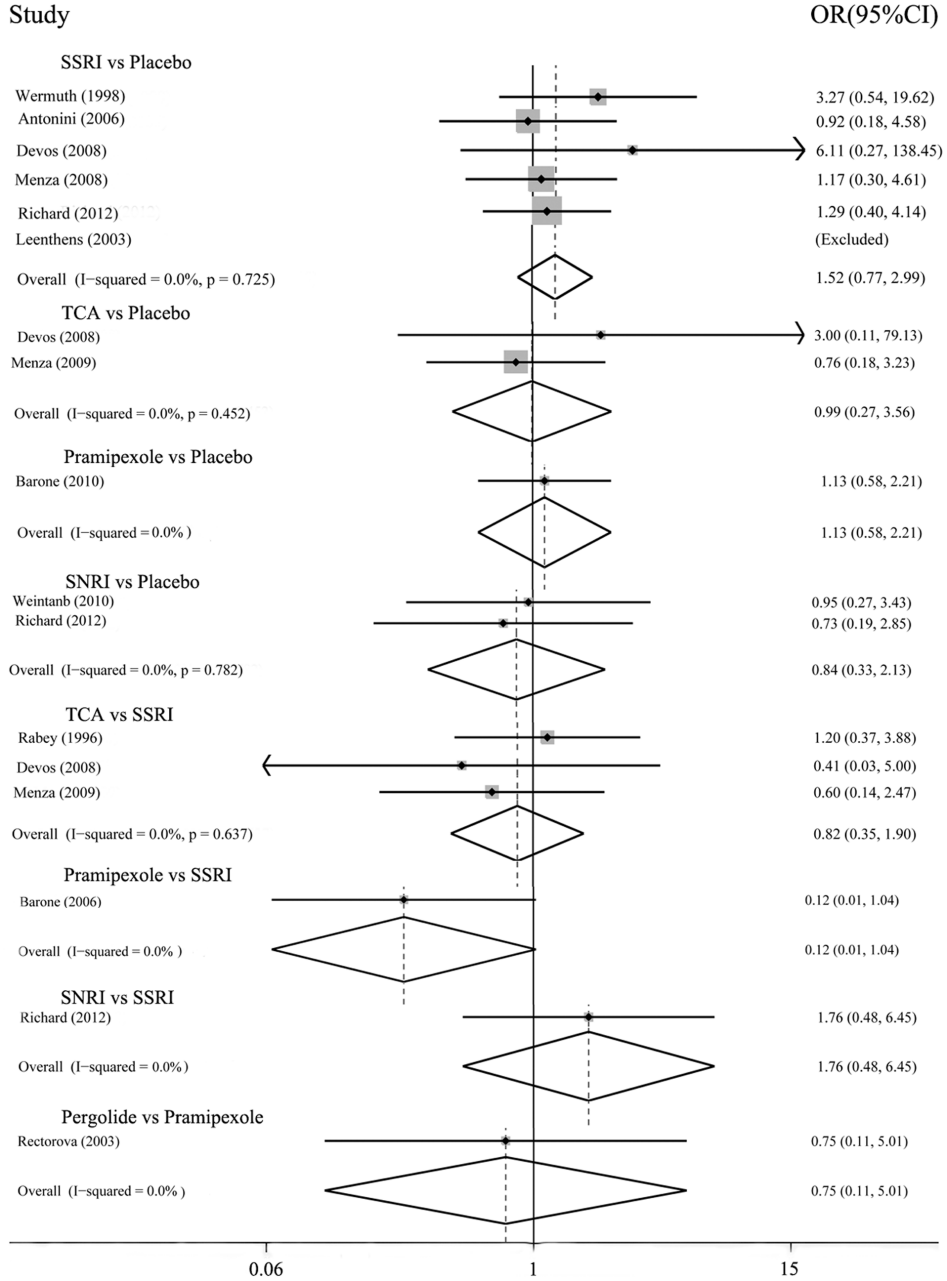
Acceptability: direct comparisons between each pair of antidepressant treatment.

In the possible pairwise comparisons between the six conditions, eight had been studied directly in one or more trials. Figure 3 and 4 showed the odds ratios for each of these direct comparisons. There was moderate heterogeneity between the three trials of SSRIs vs. TCAs (I^2^ 59.3%, P = 0.086). Three comparisons had only a single trial, heterogeneity could not be evaluated. In all other trials, there was no observed heterogeneity.

The direct comparisons (Figure 3) showed that efficacy favored TCAs over placebo, OR 4.85(1.63–14.36); results derived from other comparisons showed no statistical significance, for the 95% confidential interval of the pooled OR included 1. For dropouts (Figure 4), none of these comparisons had significance.

### Indirect comparisons

**Figure 5:**
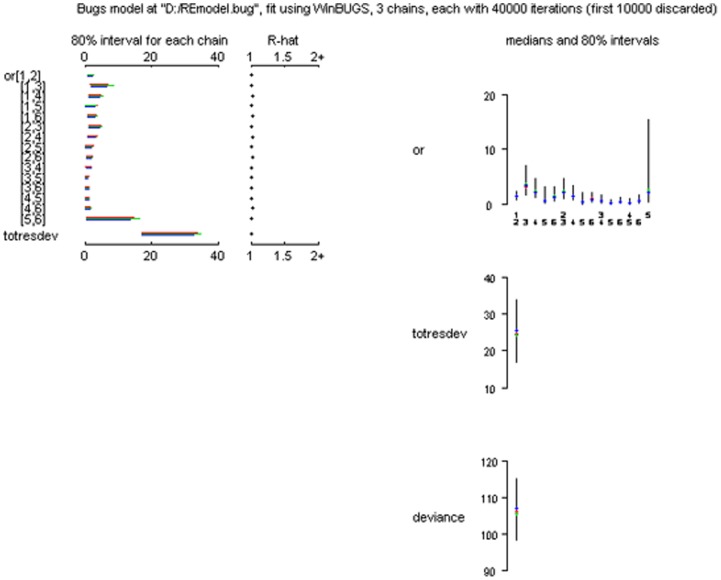
Net meta-analysis: convergence of the model, totresdev, deviance.

**Figure 6:**
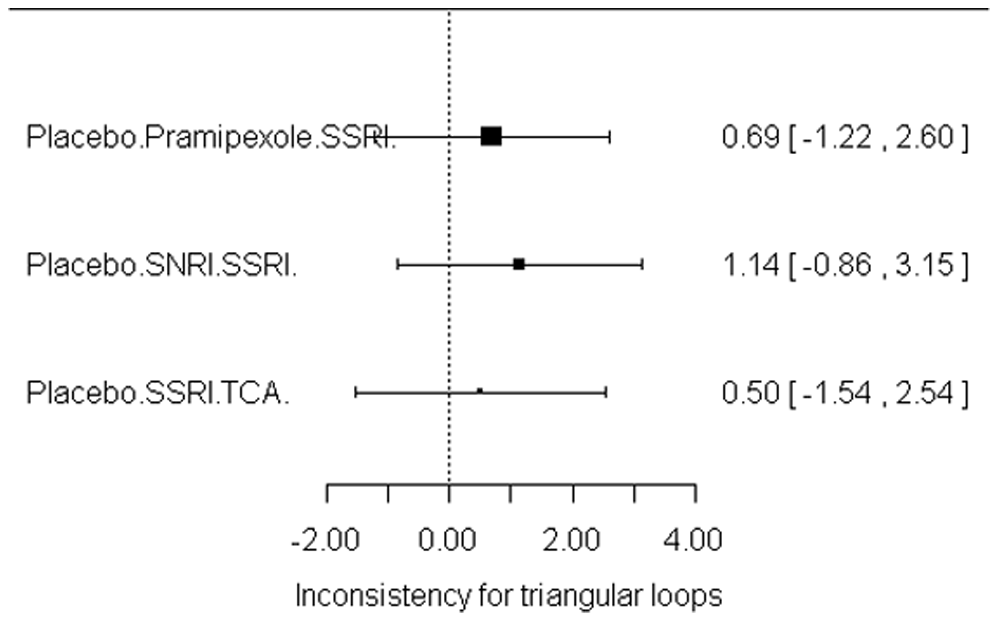
Net meta-analysis: consistency test.

Table 1 showed the results of indirect comparisons and the effect size was OR. In general, the results obtained with the direct comparisons are also retrieved in the network analysis. Of real interest are the indirect comparisons provided by the network analysis. Efficacy comparison between pramipexole and TCAs was OR 3.17(0.13–3.70); Pergolide vs. placebo 1.66(0.03–9.43); SNRIs vs. TCAs 0.64(0.07–2.48); and SNRIs vs. pramipexole 0.94(0.10–3.29). For dropouts, pramipexole vs. TCAs was OR 0.82(0.03–3.56); Pergolide vs. placebo OR 1.71(0.01–10.21); SNRIs vs. TCAs 4.32(0.29–19.09), and SNRIs vs. pramipexole 13.27(0.51–51.67). There was no statistical significance between each compared intervention. Figure 5 showed the convergence assessment of model used in the R2Winbugs and the parameter totresdev assessed model fit in our statistic method. Figure 6 showed the consistency test between direct and indirect analysis. Result suggested the consistency was good.

**Table 1:** Efficacy and acceptability of the 5 antidepressants.

SNRI	52.54(0.26–320.22)	13.27(0.51–51.67)	4.32(0.29–19.09)	2.56(0.34–9.14)	4.29(0.65–13.07)
7.63(0.15–42.88)	**Pergolide**	1.71(0.04–11.03)	1.51(0.01–9.43)	1.06(0.01–5.75)	1.71(0.01–10.21)
0.94(0.10–3.29)	0.50(0.02–2.75)	**Pramipexole**	0.82(0.03–3.56)	0.53(0.04–1.92)	0.90(0.09–2.92)
0.64(0.07–2.48)	0.58(0.01–4.03)	3.17(0.13–3.70)	**TCA**	1.00(0.19–2.83)	1.90(0.32–7.51)
1.25(0.23–3.73)	1.12(0.02–6.73)	3.44(0.48–6.29)	2.77(0.75–7.75)	**SSRI**	1.97(0.69–4.99)
1.83(0.36–5.66)	1.66(0.03–9.43)	5.84(0.72–9.87)	4.18(0.95–11.94)	1.63(0.66–3.32)	**Placebo**

Results are the ORs defined as one treatment in the table compared with underneath treatments. ORs in the lower left of the table represented efficacy, and the upper right of the table represented acceptability.

**Figure 7:**
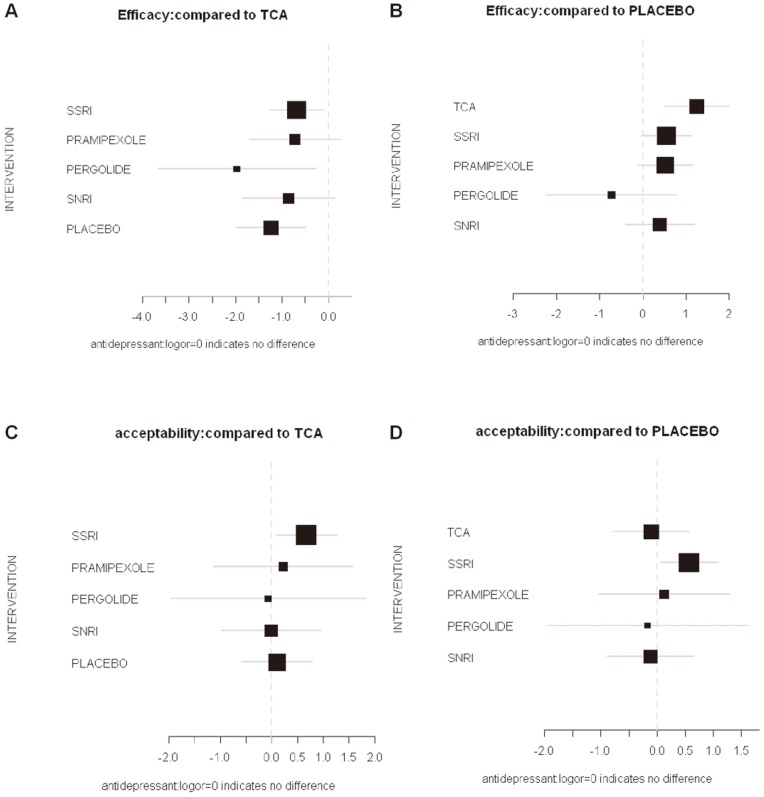
Results of net meta-analysis: using the logarithm of the odds ratios as effect size. A, B: efficacy; C, D: acceptability; A, C: TCA as the standard of comparison; B, D: Placebo as the standard of comparison.

We used the logarithm of the odds ratios as effect size to see if there were differences with the above results (Figure 7). Despite by using information from many disparate studies across several decades in different countries, the model has a low degree of incoherence (ω = 4.824827e-05). The low value suggests that the overall model was internally consistent, and could provide useful estimates of the effects of individual agents [17].

With efficacy of TCAs as the standard of comparison, the logor were: SSRIs −0.69 (95% CI −1.28– −0.10); pramipexole −0.73 (−1.71– −0.26); pergolide −1.97 (−3.67–0.27); SNRIs −0.86 (−1.86– 0.15); Placebo −1.24 (−1.99– −0.50). With Placebo as the standard of comparison, the logor were: TCAs 1.24 (0.50–1.99); SSRIs 0.55 (−0.03–1.13); Pramipexole 0.51 (−0.12–1.15); Pergolide −0.73 (−2.25–0.80); SNRIs 0.38 (−0.42–1.19).

With acceptability of TCAs as the standard of comparison, the logor were: SSRIs 0.67 (95% CI 0.08–1.27); pramipexole 0.23 (−1.13–1.58); pergolide −0.07 (−1.97–1.84); SNRIs −0.01 (−0.98–0.96); Placebo 0.11 (−0.58–0.79). With Placebo as the standard of comparison, the logor were: TCAs −0.11 (−0.79–0.58); SSRIs 0.57 (0.05–1.09); pramipexole 0.12 (−1.05–1.29); pergolide −0.17 (−1.95–1.60); SNRIs −0.12 (−0.89–0.66).

## Discussion

Our findings might help to choose antidepressants for depression in Parkinson's disease. The results showed that there were insufficient evidences to support antidepressant efficacy for SSRIs, pramipexole, pergolide and SNRIs in Parkinson's disease. In terms of response, TCAs were more efficacious than SSRIs, pramipexole, pergolide and SNRIs. In terms of acceptability, TCAs, pramipexole, pergolide and SNRIs were more tolerated than SSRIs. In fact, there were insufficient evidences to support antidepressant efficacy for SSRIs, pramipexole, pergolide and SNRIs.

It is important to note that our findings have several inherent limitations (Table 2). First, the sample size of studies was generally small and most of them were less than 50 patients. Although the effects of comparisons in some trials appeared to be quite comparable, sample size was not large enough to make any conclusions concerning the equivalence of these effects. Second, the diagnosis criteria for depression were inconsistent in Parkinson's disease. Several scales were used by different clinicians [31]. Existing diagnostic criteria for major depression may not apply well to patients with neurodegenerative diseases [32]–[33]. Third, many studies dealt with a heterogeneous population. On the one hand, PD patients might be in different stages: early or advanced. Motor fluctuations, as a common symptom in advanced Parkinson's disease, might greatly affect the occurrence and measurement of the depressive symptoms against different scales. In this research, one study had motor symptoms under control and did not experience motor fluctuations [34]; in another study by Rectorova [26], an inclusion criterion was that patients had fluctuations and/or dyskinesias; other randomized trials in the present analysis did not differentiate between patients with or without motor fluctuations. On the other hand, many studies dealt with a heterogeneous degree of depression: six studies only included patients with major depression. Fourth, duration of studies was different. Most of the trials were short term, generally lasting less than 4 months. Fifth, co-medication of studies was inconsistency. Patients in three studies were allowed to use antidepressants other than the study medication during the trial, four studies were not, and four studies were not available. Finally, in our research, we selected the (dichotomous) antidepressant response but not the continuous outcomes (standardized mean differences at endpoints) as our primary outcome, because these studies in our analysis used different scales which made it difficult to directly compare the efficacy of treatments [35]. Antidepressant response has been extensively used as the primary end point for defining improvement in many trials [36]. However, it is well known that there can be a substantial information loss when continuous outcome variables are dichotomized [37].

**Table 2:** Limitations of this meta-analysis.

Sample size of studies	Small, most of them were less than 50 patients
Evaluation criteria for depression in PD	Inconsistent, several scales were used by different clinicians, such as HDRS, MADRS, BDI and others
Heterogeneous population	PD patients might be in different stages: early or advanced; Heterogeneous degree of depression
Duration of studies	Short term, generally lasting less than 4 months
Co-medication of studies	Three studies were allowed to use antidepressants other than the study medication; four studies were not allowed; four studies were not available
Take dichotomous variable as primary outcome	There can be a substantial information loss when continuous outcome variables are dichotomized

In terms of the role of placebo, results from three studies [23]–[25] indicated that most of the benefits obtained with the active drug might derive from a placebo effect. A substantial placebo effect is apparent in Parkinson's disease (PD) [38]–[41] and depression [42]–[43]. The dopamine and opioid systems are thought to play a crucial role in the physiological response to a placebo [44]. Response to placebo in antidepressant studies has been shown to vary and has clearly increased in the past two decades, with a similar increase occurring in the fraction of patients responding to active medication as well[42]. The placebo effect in PD is related to the release of dopamine in both the dorsal and ventral striatum was found in PET studies using the dopamine D2 receptor antagonist [11C] raclopride [45]. A study about PD [46] found a larger placebo effect for the objective part of the Unified Parkinson's Disease Rating Scale (UPDRS) than that for the subjective part. Better understanding of the placebo effect can help us interpret experimental results and design more reasonable trials.

Deficits in dopaminergic, noradrenergic, and serotonergic systems have been considered to be the primary etiological factors that contribute to depression in PD [47]. Therefore, antidepressant therapy may serve as a viable treatment option for this neurological population because these drugs influence monoamine functioning. At present, antidepressants are widely used for those patients, with SSRIs being the most commonly used medications, while TCAs always being thought to have severe side effects (such as dry mouth, dry eyes, constipation, and confusion). A survey of physicians in the Parkinson Study Group found that 26% PD patients received antidepressants for depression and 51% physicians used SSRIs as their first choice [48]. A more recent Veterans Affairs database study found that 63% of patients with PD and depression were taking SSRIs, while only 7% were taking TCAs [3]. The outcome in our analysis was consistent with Okun MS et al.[49] which reminded that TCAs were not necessarily less tolerated, and SSRIs might not be as efficacious as currently perceived by practice patterns. Serotonin and norepinephrine reuptake inhibitors (SNRIs), like TCAs, act on both neurotransmitter systems but are generally better tolerated. A controlled trial of SNRIs in those patients by Richard [22] showed that both paroxetine and venlafaxine extended release significantly improved depression in subjects with PD, which is not supported by our analysis.

Pramipexole and pergolide were dopamine agonists, which were used for motor symptoms in Parkinson's disease. Few studies were designed for their antidepressant effect. Some clinical observations suggested that they might be considered for both motor and non-motor symptoms, which was good news for neurological physicians [50]. However, placebo-controlled trials are required to adequately assess the efficacy of novel antidepressant effect [51]. Barone and colleagues did the only one trial that compared pramipexole with placebo. In our research, there are insufficient evidences to support antidepressant efficacy for pramipexole and pergolide.

This meta-analysis suggests that TCAs might be the best choice when starting antidepressant treatment in Parkinson's disease because it has the most favorable balance between benefit and acceptability, followed by pramipexole and SNRIs, SSRIs might be the last one. Further randomized controlled trials are needed to check this conclusion.

This meta-analysis gives implications for future research as well. The role of antidepressant drugs should be further investigated. We need consistent definitions of depression in PD and verified scales to assess depression in those patients. We need to perform different research for different stage of patients, including patients with more severe depression (even those with suicidal ideation or psychotic symptoms) as this is a group of patients that is commonly seen in clinical practice and could benefit more from antidepressant treatment. There were still few randomized controlled trials (RCTs) of these treatment options. The small number of studies does not permit us to recommend TCAs routinely and more placebo-controlled trials are needed. In the future, our research will focus on the emergence of new studies, especially large sample studies.

## Supporting Information

Table S1 Characteristics of all the randomized controlled trials related to the analysis.
(PDF)

Checklist S1 PRISMA Checklist.
(DOC)
