# Supplementary material for: Comparative Efficacy and Acceptability of Antidepressants in Parkinson's Disease: A Network Meta-Analysis
Source: PLoS One. 2013 Oct 2;8(10):e76651. doi: 10.1371/journal.pone.0076651 (PMC3788746; doi:10.1371/journal.pone.0076651)
Supplement: Table S1 — Characteristics of all the randomized controlled trials related to the analysis. (PDF) [file pone.0076651.s001.pdf]

Study	Experimental group	Comparator	N (% male)	Mean age (age range or SD)	Duration of study	Mean duration of PD (SD)	Hoehn and Yahr stage mean (SD)	Depression Scale	Response rate	Dropout rate
Richard, et al. (2012)	paroxetine (10-40 mg/day)	1. placebo<br>2.venlafaxine (75 mg/day)	NA	NA	12 weeks	NA	NA	HDRS	Placebo: 15/34<br>Paroxetine: 23/34<br>Venlafaxine: 16/30	Placebo: 6/39<br>Paroxetine: 4/34<br>Venlafaxine: 8/42
Barone et al.(2010)	pramipexole(mean dose:2.18±0.83mg/day)	placebo	140(47.3%)	E:67.4(9.0)<br>C:66.6(9.9)	12weeks	E:4.0(4.5)<br>C:4.0(3.9)	I-III(range)	BDI	Placebo: 27/147<br>Pramipexole: 38/139	Placebo: 19/152<br>Pramipexole:20/144
Weintraub et al. (2010)	Atomoxetine (mean dose: 40mg/day)	placebo	36 (66.6%)	64.3(10.5)	8weeks	E:7.9(6.6)<br>C:5.7(5.6)	NA	IDS-C	Placebo: 2/21<br>Atomoxetine: 5/22	Placebo: 6/27<br>Atomoxetine: 6/28
Menza et al. (2009)	paroxetine CR (mean dose: 28.4 mg/day)	1.placebo<br>2. nortriptyline (mean dose: 48.5 mg/day)	52 (52%)	62.2 (8.7)	8 weeks	6.6 years	2.2	HDRS	Placebo: 4/17<br>Notriptyline:9/17<br>Paroxetine:2/18	Placebo: 6/17<br>Notriptyline: 5/17<br>Paroxetine: 7/18
Devos et al. (2008)	citalopram(20mg/day)	1. placebo<br>2.desipramine (75 mg/day)	48 (NA)	61.8 (56-68)	4 weeks	8 years	NA	MADRS	Placebo: 4/14<br>Desipramine: 11/15<br>Citalopram: 8/13	Placebo: 0/16<br>Desipramine: 1/17<br>Citalopram: 2/15
Antonini et al. (2006)	sertraline (50 mg/ day)	amitriptyline (25 mg/day)	31 (45%)	E:71.8(6.5)<br>C:68.5 (6.6)	12 weeks	E: 7.5 years<br>C: 7.3years	E: 2.0<br>C: 2.4	HDRS	Sertraline: 10/12<br>Amitripthline:8/11	Sertraline: 4/16<br>Amitripthline:4/15
Barone et al. (2006)	pramipexole (mean dose: 3.24 ± 1.3 mg/day)	sertraline (mean dose: 48.1 ± 5.9 mg/day)	67 (52%)	E:64.8(8.3)<br>C:68.1 (6.5)	12 weeks	NA	E:2.5(median)<br>C:2 (median)	HDRS	Pramipexole: 23/33<br>Sertraline: 16/33	Pramipexole: 1/33<br>Sertraline: 7/34
Rektorova et al. (2003)	pramipexole(1.5-4.5mg/day)	pergolide(1.5-4.5mg/day)	25 (61%)	E:59.7(7.7)<br>C:63.5(7.5)	8months	NA	E:2.7(0.8)<br>C:3.0(1.0)	Zung SRS<br>MADRS	Pramipexole: 8/18<br>Pergolide: 3/16	Pramipexole: 3/22<br>Pergolide: 2/19
Leentjens et al. (2003)	sertraline (25-100 mg/day)	placebo	12 (67%)	67 (7.8)	10 weeks	NA	I-IV (range)	MADRS	Placebo: 4/6<br>Sertraline: 3/6	Placebo: 0/6<br>Sertraline: 0/6

Study	Experimental group	Comparator	N (% male)	Mean age (age range or SD)	Duration of study	Mean duration of PD (SD)	Hoehn and Yahr stage mean (SD)	Depression Scale	Response rate	Dropout rate
Wermuth et al. (1998)	citalopram (10-20mg/day)	placebo	37 (43%)	64 (44-79)	6 weeks	NA	I-III (range)	HDRS	Placebo: 3/19<br>Citalopram: 2/18	Placebo: 2/19<br>Citalopram: 5/18
Rabey et al. (1996)	fluvoxamine (mean dose: 78 mg/day)	Amitriptyline (mean dose: 69mg/day)	47 (NA)	75 (NA)	16 weeks	7 years	NA	HDRS	Fluvoxamine: 12/20<br>Amitriptyline: 15/27	Fluvoxamine: 8/20<br>Amitriptyline: 12/27

PD: Parkinson's disease; MADRS: Montgomery-Asberg Depression Rating Scale; HDRS: Hamilton Depression Rating Scale; BDI: Beck Depression Inventory E: Experimental group; C: comparator-group; SD: standard deviation; NA: not available
